# Psychopathology in Adults with Autism Spectrum Disorder and Intellectual Disability

**DOI:** 10.1192/j.eurpsy.2022.177

**Published:** 2022-09-01

**Authors:** J. Mccarthy

**Affiliations:** Sussex Partnership NHS Foundation Trust, Learning Disability Service, Redhill, United Kingdom

**Keywords:** Psychopathology, intellectual disability, Autism Spectrum Disorders

## Abstract

Background: A review describing the mental health needs of adults with autism spectrum disorder (ASD) and intellectual disability (ID) published just over 10 years ago found that those with ID and ASD were more vulnerable to psychiatric disorders than adults with ID who did not have comorbid ASD (Underwood, Tsakanikos & McCarthy, 2010). Method: An overview of psychopathology presenting in adults with ID and autism including the relationship of challenging behaviours to psychiatric disorder will be summarised. A review of key papers published from 2009 to 2021 was also undertaken. The focus of the search was on assessment, diagnosis and management so as to be relevant to clinical practice. Findings: There was only one paper identified describing a new structured diagnostic tool for use with adults with ID and ASD. The majority of the new evidence focused on interventions both psychological and pharmacological. Two brief case vignettes will be presented to illustrate the diagnostic and treatment challenges. Conclusion: The past decade has produced a small increase in the evidence base on the mental health needs of adults with ID and ASD. However more evidence on effective interventions is required. Underwood L, Tsakanikos E & McCarthy J. (2010). Mental health of adults with autism spectrum disorders and intellectual disability. Current Opinion in Psychiatry, 23, 421-426.

**Disclosure:**

No significant relationships.

